# Hepatitis C virus core promotes hepatic cancer stem cell formation via β-catenin-mediated EpCAM upregulation

**DOI:** 10.1128/spectrum.02859-25

**Published:** 2026-04-08

**Authors:** Jing Yuan, Jian He, Wei Liu, Yang Luo, Yanping Li, Xianxi Zheng, Deming Deng, Fengliang Tian, Dan Nie

**Affiliations:** 1 Department of Gastroenterology, Chongqing Traditional Chinese Medicine Hospital, Chongqing, China; 2 Department of Preventive Medicine, Chongqing Tongnan Hospital of Traditional Chinese Medicine 117933 , Chongqing, China; University of Manitoba, Winnipeg, Canada

**Keywords:** hepatitis C virus core, Wnt/β-catenin signaling, hepatic cancer stem cells, cell differentiation

## Abstract

Hepatitis C virus (HCV) infection is a major cause of hepatocellular carcinoma (HCC). However, how HCV promotes hepatic progenitor cell (HPC) differentiation into hepatic cancer stem cells (HCSCs) remains unclear, particularly the role of β-catenin signaling and epithelial cell adhesion molecule (EpCAM) regulation in this process. We used mouse HPCs (HP14.5 and HP14.5-Core) and human HCC cell lines (Huh7, HepG2) to investigate HCV core function. Cells were transduced with AdCore, AdGFP, or shRNAs targeting EpCAM or β-catenin. Functional properties were evaluated using spheroid formation, ICG uptake, colony formation, migration, invasion, and *in vivo* tumorigenesis assays. EpCAM promoter activity and Wnt/β-catenin signaling were examined by luciferase reporter assays. Protein expression was measured by RT-PCR and Western blotting, while immunofluorescence and co-immunoprecipitation were used to analyze HCV core-β-catenin interactions. HCV core expression increased spheroid number, reduced ICG uptake, and accelerated tumor growth in xenograft and orthotopic mouse models, consistent with HPC differentiation toward HCSCs. EpCAM, CD133, CD44, and CD90 were strongly upregulated, whereas EpCAM silencing suppressed proliferation, migration, and invasion. Reporter assays revealed that HCV core enhanced EpCAM promoter activity and activated Wnt/β-catenin signaling. Immunofluorescence demonstrated β-catenin nuclear translocation, and co-immunoprecipitation confirmed direct interaction between HCV core and β-catenin. Knockdown of β-catenin reduced EpCAM, c-Myc, and cyclin D1 expression, diminished spheroid formation, restored ICG uptake, and significantly impaired tumorigenesis *in vivo*. The HCV core-β-catenin-EpCAM axis drives the conversion of HPCs into HCSCs. This pathway is a central mechanism in HCV-related HCC.

**IMPORTANCE:**

HCV core protein directly interacts with β-catenin, driving its nuclear translocation and activating EpCAM expression. This promotes HPC differentiation into HCSCs, marked by increased spheroid formation, migration, invasion, and tumorigenesis. Silencing EpCAM or β-catenin reverses these effects, confirming that the HCV core-β-catenin-EpCAM axis is a key pathway in HCV-related HCC.

## INTRODUCTION

Hepatocellular carcinoma (HCC) stands as the world’s third most lethal malignancy (1). Even with therapeutic breakthroughs, outcomes remain dismal, as most patients arrive at clinics with late-stage disease (2). Chronic hepatitis C virus (HCV) infection is a risk factor for HCC, increasing the likelihood of malignant transformation by 20–30 times compared with uninfected individuals (3–7). Emerging evidence suggests that HCV contributes to hepatocarcinogenesis not only through chronic inflammation but also by influencing hepatic progenitor cells (HPCs), which can acquire properties of hepatic cancer stem cells (HCSCs) (4). However, the molecular mechanisms underlying this process are ambiguous.

HCV belongs to the *Flaviviridae* family of viruses. Its core protein, a structural component with RNA-binding activity, has been implicated in regulating several host signaling pathways (8–14). The HCV core protein interacts with transcription factors such as NF-κB and Snail (9–11), modulates growth factor signaling (12, 13), and promotes metastasis through pathways including MAPK/ERK and epithelial-mesenchymal transition (EMT) (15–17). Notably, increasing evidence indicates that the HCV core may directly regulate pathways including Wnt/β-catenin pathway for cell proliferation and tumor progression (14, 18).

The Wnt/β-catenin signaling aids in embryonic development and stem cell biology and controls the degradation of β-catenin protein which is important for cell adhesion and activation of proliferation genes (19, 20). However, the unnecessary pathway activation induces β-catenin accumulation in the nucleus. This, in turn, drives transcription of c-Myc, cyclin D1, and epithelial cell adhesion molecule (EpCAM) which are considered oncogenes (21–24). In HCC, β-catenin activation has been linked to tumor initiation, self-renewal of HCSCs, and immune evasion (25–28). Among its downstream targets, EpCAM functions as both a cell adhesion molecule and an oncogenic signaling protein involved in proliferation, stemness, migration, and EMT (29–33). EpCAM has also been proposed as a diagnostic biomarker and pivotal target for therapy (34–36). Previous studies have suggested that HCV core may enhance tumor aggressiveness by activating Wnt/β-catenin signaling and inducing EMT (37, 38). Yet, the precise mechanism by which HCV core drives HPC differentiation toward HCSCs and the role of EpCAM in this process remain to be fully elucidated.

In this study, we investigated whether the HCV core protein promotes the differentiation of HPCs into HCSCs through interaction with β-catenin and subsequent activation of Wnt/β-catenin signaling. Specifically, we examined how HCV core influences EpCAM expression, assessed its impact on HPC differentiation and tumorigenic potential, and determined the role of β-catenin in mediating these effects.

## MATERIALS AND METHODS

### Cell culture

The HCC cell lines Huh7 and HepG2 were obtained from ATCC, and the mouse mesenchymal cell line C3H10T1/2 was purchased from the Chinese Academy of Sciences. Cells were cultured in DMEM medium containing fetal bovine serum at concentration of 10% with additional elements such as penicillin and streptomycin (100 µg/mL each). Mouse hepatic progenitor cell lines HP14.5 and HP14.5-Core were maintained as previously described (39). All these cells were maintained in a standard incubator (37°C and 5% CO₂) for experiments.

### Adenoviruses, plasmids, and siRNA

The adenoviruses AdCore and AdGFP and shRNA adenoviruses AdR-shEpCAM, AdR-shβ-catenin, AdR-shCore, and AdR-shControl were generated using the AdEasy system (40). A scrambled shRNA control (AdR-shControl) expressing RFP was used as control. Expression plasmid pcDNA3.1-EpCAM was obtained from Addgene (Cambridge, MA, USA) as described previously (41).

For infection experiments, HP14.5, C3H10T1/2, HepG2, and Huh7 cells were infected with AdCore or AdGFP at a multiplicity of infection (MOI 50) for 24 h and then maintained in complete medium for an additional 24–48 h before being harvested for protein or RNA analysis. Stable lines expressing GFP (HP14.5-GFP) or HCV core (HP14.5-Core) were also used directly in some assays. For overexpression experiments, HP14.5-Core cells were transfected with pcDNA3.1-EpCAM using Lipofectamine 2000 (Invitrogen) and collected 48 h post-transfection for analysis. Finally, for silencing experiments, HP14.5-GFP or HP14.5-Core cells were infected with AdR-shCore, AdR-shEpCAM, or AdR-shβ-catenin (MOI 50) for 24 h and maintained in complete medium for 48–72 h before harvest. AdR-shControl was used as a negative control.

### siRNA-mediated β-catenin silencing

HP14.5 cells were targeted for silencing β-catenin by transfecting them with siRNAs (si-β-catenin-1 and si-β-catenin-2; GenePharma, Shanghai, China) or a scrambled siRNA control using Lipofectamine 2000 (Invitrogen) as stated in the protocol. After 48 h, the cells were processed to evaluate the knockdown efficiency.

### Hepatic differentiation induction

HP14.5 cells were seeded at ~60% confluence and infected with AdGFP or AdCore for 24 h at MOI 50. After infection, the medium was replaced with differentiation medium consisting of DMEM supplemented with 10⁻⁶ M dexamethasone and 1% DMSO. Cells were maintained in this medium for the indicated times (0, 7, and 15 days), with medium renewed every 2–3 days and harvested at indicated times.

### RNA isolation and quantitative RT-PCR

RNA was extracted from cells using the standard TRIzol reagent (Invitrogen, Cat. No. 15596026) and concentration of final RNA were determined with a NanoDrop. RNA (750 μg) was processed to prepare cDNA using the reverse transcription kit (TaKaRa, Cat. No. RR037A). Quantitative PCR was performed using SYBR Premix Ex Taq II (TaKaRa, Cat. No. RR820A) on an ABI 7500 Real-Time PCR System. Primer sequences for *Albumin* (*Alb), Cytokeratin 18 (CK18), EpCAM, CD133, CD44, CD90*, and *GAPDH* are listed in Table 1. Gene expression was calculated using the 2^ΔΔCt^ method with respect to GAPDH.

**TABLE 1:** Sequence of nucleotides in primers for RT-qPCR

Gene	Forward	Reverse
*Alb*	AGTGAGGAGGAGGACATCAG	TCTGGTCTCAGTGTGGTTGA
*CK18*	CAGGAGTTTGAGGAGGACAG	ATGGTGCTTGTGGTCTTCAC
*EpCAM*	AGCTCAGGAAGAATGTGGTG	CTTGGAGTCAAAGTCCTGGA
*CD133*	TCTTTGGATTCCTGCTGTTG	CAGGTAGTTGTTGCCAGTGA
*CD44*	GACTCCAGTCATAGTACAACGGA	TTGCTCCACCTTCTTGACTC
*CD90*	TGGAGACGCTGGTGTTACTT	CCTTCTGAGGAGGTTGTTGA
*GAPDH*	AAGGTCATCCCAGAGCTGAA	CTGCTTCACCACCTTCTTGA

### Western blot analysis

Cells were lysed in RIPA buffer supplemented with protease and phosphatase inhibitors (Beyotime, Cat. No. P0013B). The protein values were determined by running the standard BCA assay (Thermo Fisher Scientific). Afterward, equal amounts of proteins (30 μg) were separated by SDS-PAGE which were then promptly shifted to PVDF membranes, and then these membranes were blocked for any non-specificity and undesirable binding with 5% milk in TBST which acted as a blocking agent for 1 h at room temperature. The specific binding sites on these membranes were exposed, and membranes were incubated with desired primary antibodies overnight at 4°C. The list of antibodies with dilutions and catalogs numbers were: EpCAM (Abcam, ab213500, 1:250), CD133 (Abcam, ab222782, 1:1,350), CD44 (Bioworld, BS6825, 1:20), CD90 (CST, #13801, 1:550), HCV core (Abcam, ab2740, 1:300), c-Myc (Santa Cruz, sc-764, 1:25), cyclin D1 (CST, #7918, 1:950), β-catenin (Santa Cruz, sc-7199, 1:450), VEGF (Santa Cruz sc-7269, 1:50), active β catenin (Cell signaling, #D13A1, 1:250), and GAPDH (CST, #2118, 1:350). On the next morning, all the membranes were retrieved and washed with washing buffer to remove the free floating antibody and then were incubated with secondary antibodies (Anti-mouse, Cat # A16066, 1:5,000 and Anti-Rabbit, Cat # 31466, 1:3,000) for 1 h on bench at room temperature. The membranes after washing were treated with enhanced chemiluminescence (ECL, Thermo Fisher) for visualization of bands which were then quantified by ImageJ.

### Immunofluorescence staining

Cells were grown at 1 × 10^6^ cells in each well on a glass coverslips in a 12 well plate. The cells were fixed in formalin and permeabilized with Triton X-100 for 10 min to allow antibody to penetrate. The cells were washed briefly and blocked with 2% goat serum for 45 min and then incubated overnight at 4°C with primary antibodies against β-catenin (CST, #9562, 1:350), anti-HCV core (Abcam, ab2740), and EpCAM (Abcam, ab213500). On the next day, cells were gently washed to remove primary antibody and incubated with secondary antibodies (goat anti-rabbit, 1:500) for 1 h at room temperature and mounted with antifade medium. For dual staining of IF, we mixed β-catenin and anti-HCV core antibodies together at primary and secondary stage of antibodies. Images were acquired on a fluorescence microscope using identical exposure settings for all groups.

### Co-immunoprecipitation assay

HP14.5 cells were infected with AdCore or AdGFP (MOI 50) for 24 h and maintained for an additional 24 h. Cells were washed in ice-cold PBS and lysed on ice in RIPA buffer (Beyotime) supplemented with protease/phosphatase inhibitors for 30 min with gentle mixing. Lysates were cleared by centrifugation (12,000 × *g*, 15 min, 4°C), and protein concentration was determined by BCA. For each IP, 0.5–1.0 mg total protein was adjusted to 500 μL with lysis buffer and pre-cleared with 20 μL protein G agarose (Millipore) for 1 h at 4°C.

Pre-cleared supernatants were incubated overnight at 4°C with 2–5 μg antibody: anti-HCV core (Abcam, ab2740), anti-β-catenin (CST#12475, 1:250), or species-matched normal IgG (negative control). Immune complexes were captured with 30 μL protein G agarose for 2 h at 4°C and then washed 4–5× with ice-cold RIPA buffer. Bound proteins were eluted by boiling in 2× Laemmli buffer (5 min), separated by 10%–12% SDS-PAGE, and transferred to PVDF membranes. Reciprocal co-IPs were performed in parallel (anti-core IP immunoblotted for β-catenin; anti-β-catenin IP immunoblotted for core). Input lysate (5%–10%) and IgG IP were included as controls. Finally, the blots were incubated with ECL.

### Dual-luciferase reporter assay

HP14.5 cells at a density of 0.4 × 10⁶ cells were plated in 24-well plates and transfected with either the pGL3-EpCAM promoter reporter (containing a 2.2-kb EpCAM promoter/enhancer fragment with two functional Tcf binding elements) or the empty pGL3-Basic vector (Promega) using Lipofectamine 2000 (Invitrogen) as shown previously (42). After 16 h, cells were infected with AdCore or AdGFP (MOI 50) and cultured for an additional 24 h. For promoter assays under EpCAM knockdown, HP14.5-GFP and HP14.5-Core cells were first transduced with AdR-shEpCAM or AdR-shControl (MOI 50) for 24 h and maintained in complete medium for a further 48 h before plasmid transfection. Cells were harvested after 24 h of transfection procedure, and luciferase activity was measured with Dual-Luciferase Reporter Assay System (Promega, Cat. No. E1910) on a luminometer. Renilla luciferase plasmid (pRL-TK, Promega) was co-transfected as an internal control for transfection efficiency. Results were expressed as relative firefly luciferase activity normalized to Renilla and pGL3-Basic control.

### TOP/FOP luciferase reporter assay

HP14.5 cells (0.3 × 10⁶/well, 24-well plates) were transfected with TOPflash (wild-type TCF motifs) or FOPflash (mutated motifs; Millipore) plasmids using Lipofectamine 2000 (Invitrogen). A Renilla luciferase construct (pRL-TK, Promega) served as a calibration control. Sixteen hours post-transfection, cells were challenged with AdCore or AdGFP (MOI 50); untreated cells provided a baseline reference. After 24 h, lysates were harvested, and luciferase signals quantified with the Dual-Luciferase Reporter System (Promega, E1910). Results were conveyed as TOP/FOP ratios normalized to Renilla activity.

### Colony formation assay

HP14.5-GFP and HP14.5-Core cells were exposed to AdR-shEpCAM or to the control virus (AdR-shControl) at an MOI of 50 for 24 h. After treatment, cultures were maintained in medium containing blasticidin (5 µg/mL; Invitrogen) for about 3 weeks, with fresh medium added every 2–3 days. To maintain sustained knockdown throughout the selection period, we employed a multiple infection strategy. After the initial 24 h infection with AdR-shEpCAM or AdR-shControl, cells were cultured in complete medium. At regular intervals during the subsequent cultivation period (every 5–7 days), cells were re-infected with the respective adenoviral vectors at the same MOI of 50. This iterative infection approach was necessary because adenoviruses do not integrate into the cell genome and, therefore, cannot achieve long-term stable expression. By repeatedly introducing fresh viral particles, we ensured continuous shRNA expression, thereby maintaining sustained knockdown of target genes (EpCAM and β-catenin) throughout the 3-week selection period. The MOI was strictly maintained at 50 for each infection to ensure consistent infection efficiency and preserve cell viability. At the end of selection, surviving colonies were fixed in 4% paraformaldehyde for 15 min and stained with 0.5% crystal violet for 30 min. Cells with aggregates of more than 50 cells were considered colony, and the mean colony number was calculated from at least three independent experiments. Colony counts were normalized to the control group for comparison.

### Cell migration and invasion assay

The invasion and migration of cells were carefully monitored and evaluated using Transwell chambers (8-µm pore size; Corning). For migration, 5 × 10⁴ HP14.5-GFP or HP14.5-Core cells transduced with AdR-shEpCAM or AdR-shControl were seeded on the top chamber in serum-free medium. The chamber below the top one was filled with 10% cell culture medium as a chemoattractant. After 24 h, the migrated cells on the lower surface were fixed with cold formalin and stained with 0.1% crystal violet.

For invasion, the inserts were precoated with Matrigel, and 1 × 10⁵ cells were plated in the same manner. After 48 h, invading cells were fixed, stained, and imaged. For both assays, cells from at least five random microscopic fields were counted, and the mean number of invaded cells was calculated from three independent experiments.

### Spheroid formation assay

HP14.5-GFP and HP14.5-Core cells were seeded in 6-well plates (1 × 10³ cells/well) and cultured in serum-free DMEM/F12 medium consisting of growth factors and other supplements such as B27, EGF (20 ng/mL), and bFGF (20 ng/mL). Where indicated, cells were first transduced with AdR-shβ-catenin or AdR-shControl (MOI 50) for 24 h and maintained for 48–72 h before spheroid initiation. The media was changed every alternate day to support optimal growth. After 14 days, spheroids larger than 50 μm were counted, and representative images were recorded. Results were expressed as the mean number of spheroids per field, and values for HP14.5-GFP were set to 100% for relative comparison.

### Indocyanine green uptake

HP14.5-GFP and HP14.5-Core cells were cultured in differentiation medium containing dexamethasone (10⁻⁶ M) and 2% DMSO for 14 days. Where indicated, cells were first transduced with AdR-shβ-catenin or AdR-shControl (MOI 50) for 24 h and maintained for 48–72 h before induction. Cells were then washed and incubated with DMEM containing freshly prepared ICG (1 mg/mL; Sigma-Aldrich, Cat. No. I2633) for 1 h at 37°C. After incubation, cells were washed and ICG retention was visualized by light microscopy. For quantification, ICG-positive staining was analyzed from at least 10 non-overlapping fields. Uptake was expressed as the relative percentage compared to HP14.5-GFP cells, which were set at 100%.

### *In vivo* tumor formation

All animal-related procedures were approved by Ethics Committee of Chongqing Hospital of Traditional Chinese Medicine (CH2024_TY998902). The subcutaneous xenograft model was established by subcutaneously injecting HP14.5-GFP (vector control) and HP14.5-Core cells (1 × 10⁵ cells in 50 μL PBS) into the flanks of athymic nude mice (*n* = 5 per group). Tumor length (*L*) and width (*W*) were measured weekly for 5 weeks with calipers, and volume was calculated as *V* = (*L* × *W* × *W*)/2. Mice were euthanized at week 5, and tumors were excised for gross documentation. Mice were humanely sacrificed under deep anesthesia with isoflurane, followed by cervical dislocation. HP14.5-GFP and HP14.5-Core cells (1 × 10⁵ cells in 30–35 μL PBS) were injected into the left liver lobes of nude mice (*n* = 5 per group) and sacrificed after 8 weeks, to collect livers for gross imaging and H&E histological evaluation. For knockdown studies, HP14.5-Core cells were transduced with AdR-shβ-catenin or control shRNA (AdR-shControl) for 24 h before implantation. Cells (1 × 10⁵ per injection in 50 μL PBS) were implanted into the left liver lobe of nude mice (*n* = 5 per group). The growth of induced tumors was observed for 8 weeks, after which mice were humanely sacrificed as stated above and livers were excised for further experimental procedures.

### Immunohistochemical staining

The liver tissues were fixed in formalin and embedded which were cut into 4-µm sections. Sections were cleared in xylene and sequentially rehydrated through descending ethanol gradients to distilled water. Antigen retrieval was performed in a beaker containing 10 mM sodium citrate buffer in an oven for 10 min, and endogenous peroxidase activity was blocked with 3% hydrogen peroxide for 15 min. Sections were incubated overnight at 4°C with primary antibodies against HCV Core (Abcam, ab2740), EpCAM (Thermo Fisher, #11-5791-82, 1:120 dilution), CD133 (Proteintech, 18470-1-AP, 1:50 dilution), CD44 (Thermo Fisher, #14-0441-82, 1:60 dilution), or CD90 (Abcam, ab133350, 1:750 dilution). After washing, slides were incubated with anti-mouse (Thermo Fisher, #61-6520, dilution 1:150) and anti-rabbit secondary antibodies (Thermo Fisher, **#**31464, dilution 1:350) for 30 min, developed with DAB substrate, and counterstained with hematoxylin. The slides were dehydrated and mounted, and images were taken with a microscope.

### Statistical analyses

All assays were executed in triplicate. Data are depicted as mean ± SD and interpreted with SPSS 24.0 (IBM) and GraphPad Prism. Two-group contrasts employed Student’s *t*-test, while multiple-group variances were interrogated using one-way ANOVA.

## RESULTS

### HCV core promotes the differentiation of HPCs into HCSCs

**Fig 1:**
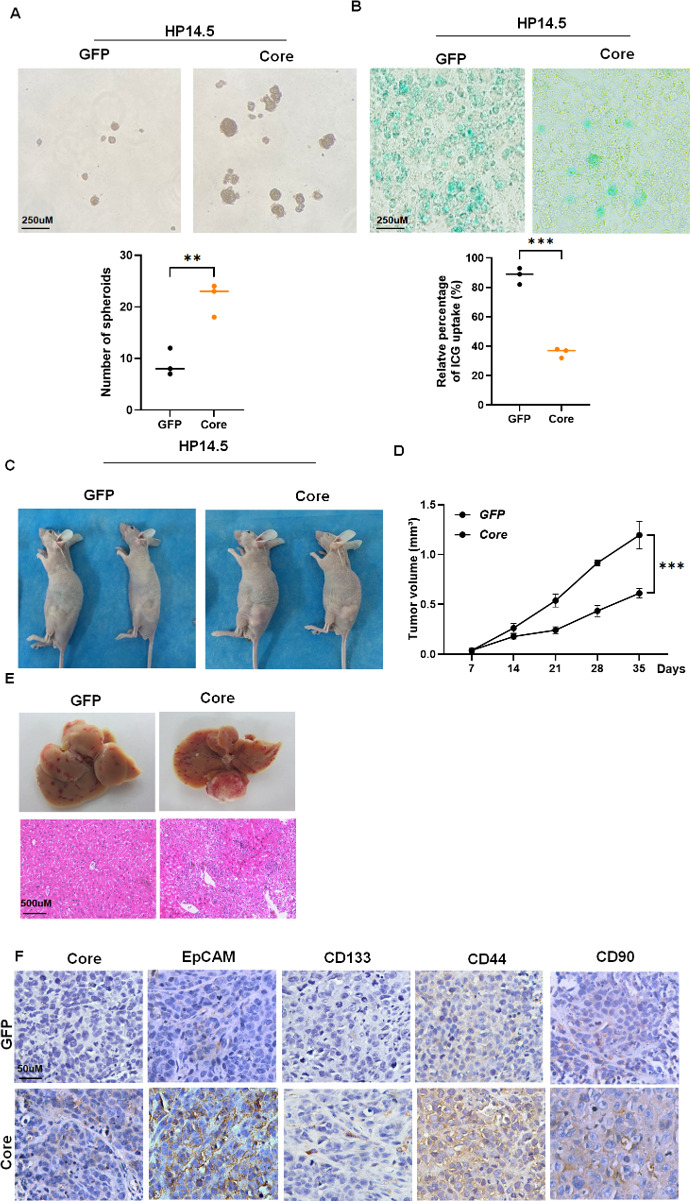
HCV core promotes differentiation of HPCs into HCSCs. (**A**) Sphere formation in HP14.5-GFP and HP14.5-Core cells after 2 weeks in spheroid culture (*n* = 3). (**B**) ICG uptake assay in HP14.5-GFP and HP14.5-Core cells treated with Dex/DMSO for 14 days (*n* = 3). (**C**) Representative xenograft tumors from subcutaneous injection of HP14.5 or HP14.5-Core cells (*n* = 5). (**D**) Tumor volume growth curve over 5 weeks (*n* = 5). ***P < 0.001 (HP14.5-GFP vs HP14.5-Core). (**E**) Representative liver images and H&E staining following orthotopic implantation of HP14.5-GFP or HP14.5-Core cells (*n* = 5). (**F**) IHC staining for HCV core, EpCAM, CD133, CD44, and CD90 in liver tissues (*n* = 5). **P < 0.01.

We first examined whether HCV core expression drives HPCs toward cancer stem-like properties. In serum-free culture, HP14.5 cells expressing HCV core formed significantly more spheroids than control cells after 2 weeks, indicating enhanced self-renewal capacity (Fig. 1A). Conversely, ICG uptake, a marker of mature hepatocyte function, was reduced in HCV core-expressing cells, suggesting impaired differentiation toward mature hepatic lineages (Fig. 1B). We next assessed tumorigenic potential *in vivo*. Subcutaneous xenografts demonstrated accelerated tumor growth in HCV core-expressing cells (Fig. 1C and D). Orthotopic implantation into the liver confirmed these findings, with core-expressing cells producing larger and more invasive tumors and histological examination revealed poorly differentiated lesions (Fig. 1E). Finally, IHC showed elevated expression of HCSC markers EpCAM, CD133, CD44, and CD90 in tumors derived from HCV core-expressing cells (Fig. 1F). Overall, HCV core promotes the appearance of cancer stem-like features in HPCs and enhances their tumorigenic capacity.

### HCV core impairs hepatic differentiation and upregulates cancer stem cell markers

**Fig 2:**
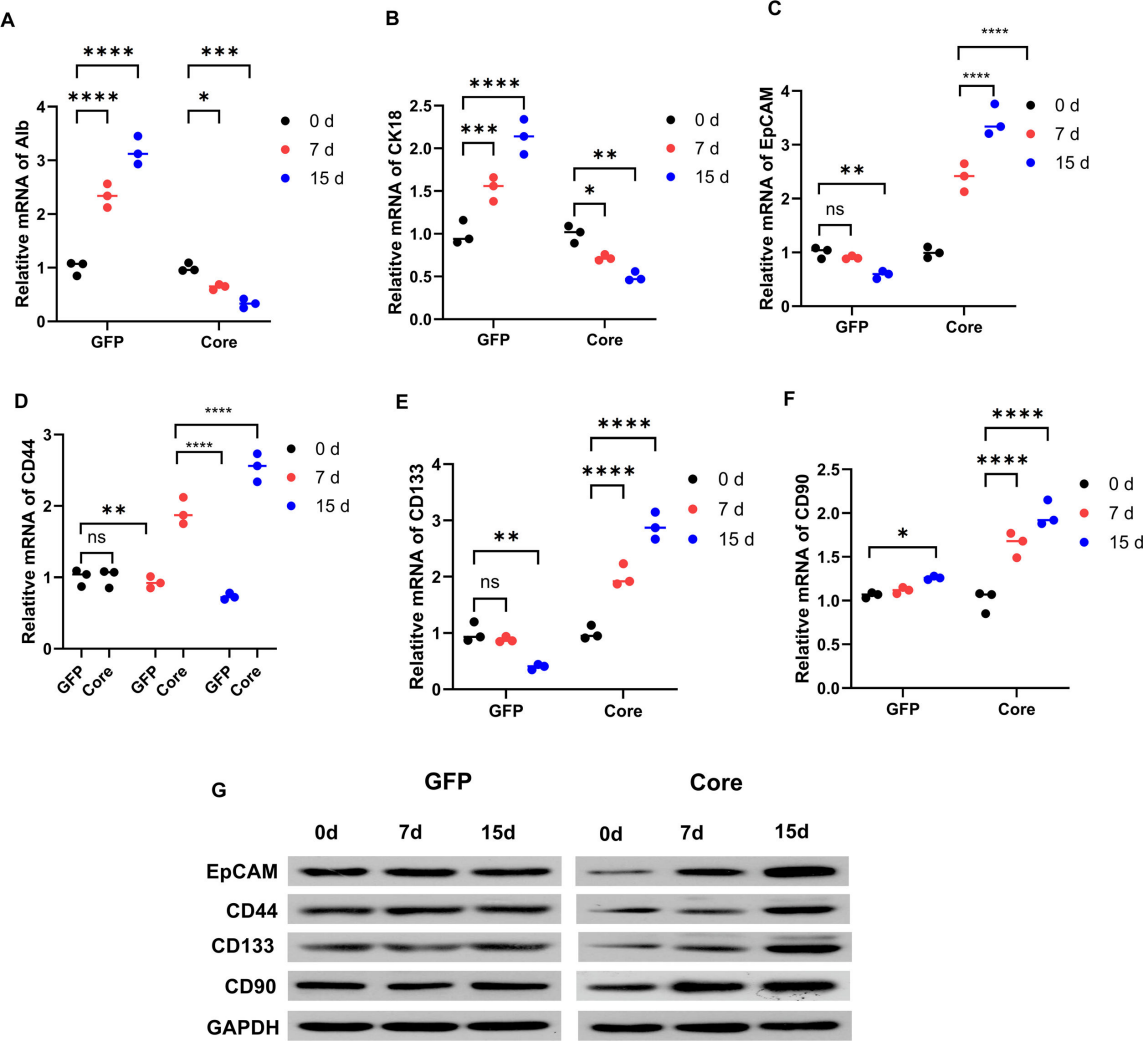
HCV core impairs hepatic differentiation and upregulates HCSC markers. HP14.5 cells were infected with AdGFP or AdCore for 24 h, treated with 10^−^⁶ M Dex and 2% DMSO for the indicated times (0, 7, 15 days), and analyzed (**A and B**) mRNA levels of hepatocyte differentiation markers Alb and CK18 increased in GFP controls but were suppressed in core-expressing cells (*n* = 3). *P < 0.05, ***P < 0.001, ****P < 0.0001. (**C–F**) mRNA levels of HCSC markers EpCAM, CD44, CD133, and CD90 were upregulated in core-expressing cells compared with controls (*n* = 3). ns: *P* > 0.05, *P < 0.05, **P < 0.01, ***P < 0.001, ****P < 0.0001. (**G**) Western blot validation of HCSC marker expression (*n* = 3).

Loss of hepatic identity and gain of stemness are key steps in malignant transformation; therefore, we next examined how HCV core affected the differentiation status of HP14.5 cells. RT-qPCR showed that the hepatocyte lineage genes Alb and CK18 increased over time (0, 7, 15 days) in GFP control cells, consistent with progression toward mature hepatocyte differentiation. In contrast, their expression was suppressed and nearly abolished by day 7 in HCV core-expressing cells (Fig. 2A and B), indicating that the core protein blocks hepatic maturation. We then analyzed HCSC markers. Core expression enhanced EpCAM, CD44, CD133, and CD90 at mRNA (Fig. 2C through F) and protein levels (Fig. 2G), and their levels continued to rise with prolonged induction time. In conclusion, HCV core prevents normal hepatic differentiation while driving the acquisition of HCSC-associated marker expression.

### HCV core upregulates EpCAM expression and EpCAM knockdown attenuates HCV core-induced proliferation, migration, and invasion

**Fig 3:**
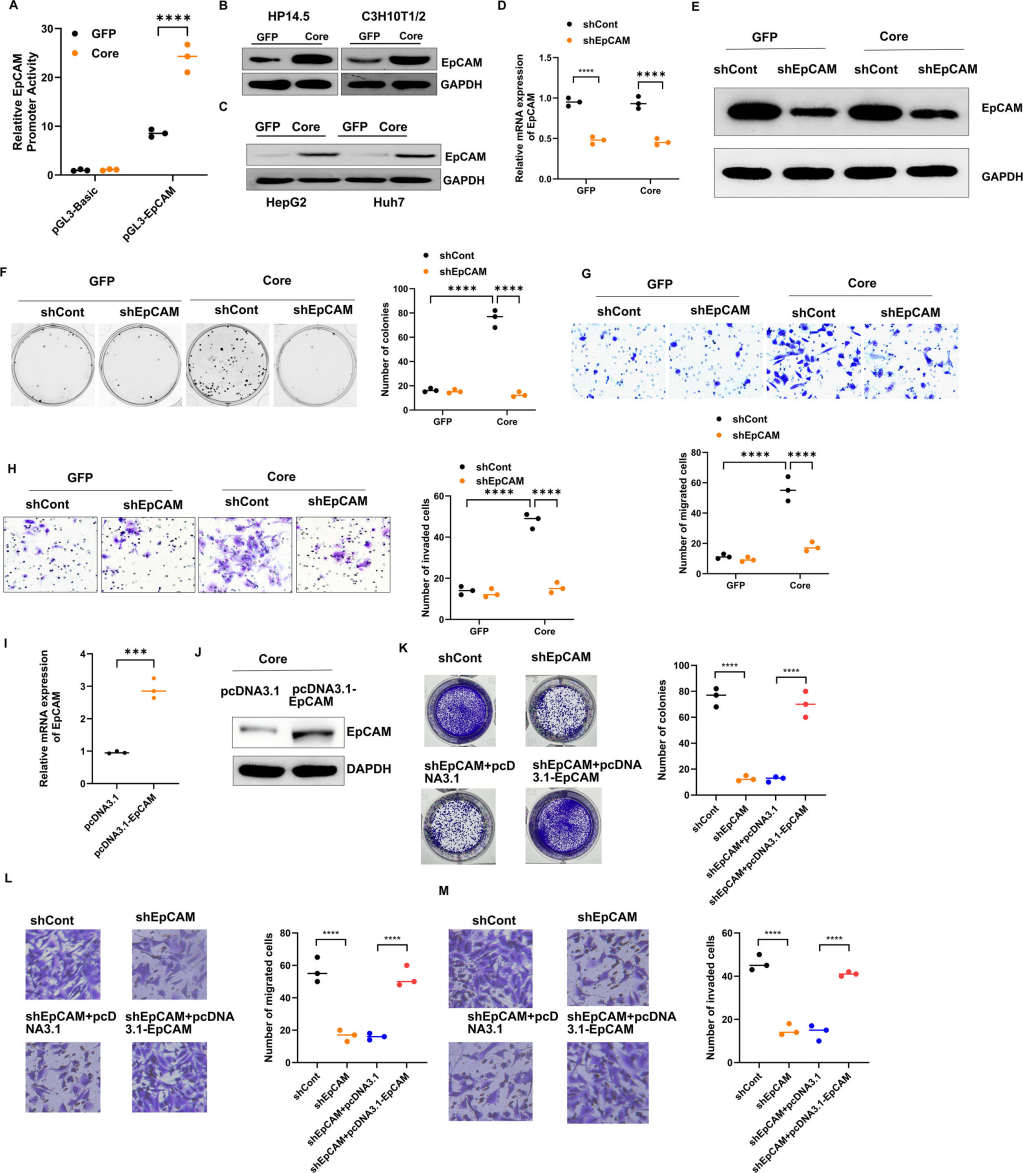
HCV core upregulates EpCAM and promotes proliferation, migration, and invasion in an EpCAM-dependent manner and EpCAM overexpression restores the tumorigenic and invasive properties lost due to EpCAM silencing in HP14.5-Core cells. (**A**) Luciferase assay showed EpCAM promoter activity in HP14.5 cells infected with AdCore or AdGFP (*n* = 3). Data are normalized to pGL3-Basic controls. (**B and C**) Western blot analysis of EpCAM expression in AdCore- or AdGFP-infected HP14.5, C3H10T1/2, HepG2, and Huh7 cells (*n* = 3). (**D and E**) RT-qPCR and western blot analysis of EpCAM expression in HP14.5-GFP and HP14.5-Core cells transduced with shControl or shEpCAM. (**F**) Colony formation assay in HP14.5-GFP and HP14.5-Core cells transduced with shControl or shEpCAM and selected with blasticidin for 3 weeks (*n* = 3); colonies were stained with crystal violet and quantified. (**G and H**) Transwell assays showed that EpCAM knockdown reduced migration and invasion of HP14.5-Core cells compared with shControl (*n* = 3). (**I and J**) RT-qPCR and western blot analysis of EpCAM expression in HP14.5-Core cells transduced with pcDNA3.1 or pcDNA3.1-EpCAM. (**K**) Colony formation assay in HP14.5-Core cells transduced with shEpCAM and pcDNA3.1-EpCAM (*n* = 3); colonies were stained with crystal violet and quantified. (**L and M**) Transwell assays detected the cell migration and invasion of HP14.5-Core cells transfected with transduced with shEpCAM and pcDNA3.1-EpCAM (*n* = 3). ***P < 0.001, ****P < 0.0001.

EpCAM has been linked to hepatic cancer stem cell features, and co-expression with AFP defines a subtype of HCC associated with poor prognosis and high invasiveness (43). To investigate whether HCV core regulates EpCAM, we first assessed its promoter activity. HP14.5 cells transfected with the pGL3-EpCAM reporter showed a marked increase in luciferase activity following AdCore infection compared with AdGFP controls (Fig. 3A). Consistently, Western blot analysis demonstrated elevated EpCAM protein levels in AdCore-infected HP14.5 and C3H10T1/2 cells (Fig. 3B), as well as in human HCC cell lines HepG2 and Huh7 (Fig. 3C) indicating that HCV core activated EpCAM at both the promoter (Fig. 3A) and protein levels (Fig. 3B and C). We next examined whether EpCAM is functionally required for HCV core-mediated phenotypes. HP14.5-GFP and HP14.5-Core cells were transduced with Ad-shEpCAM or shControl, and RT-qPCR and Western blot analyses confirmed that EpCAM mRNA and protein expression were significantly reduced in both HP14.5-GFP and HP14.5-Core cells following infection with Ad-shEpCAM (Fig. 3D and E). In HP14.5-Core cells, EpCAM knockdown reduced both the number and size of blasticidin-resistant colonies, while having little effect in GFP controls (Fig. 3F). Likewise, transwell assays demonstrated that EpCAM silencing significantly suppressed migration (Fig. 3G) and invasion (Fig. 3H) in core-expressing cells, but not in GFP cells. In addition, we also overexpressed EpCAM in HP14.5-Core cells by transfecting pcDNA3.1-EpCAM, and RT-qPCR and Western blot analyses confirmed successful EpCAM overexpression (Fig. 3I and J). EpCAM overexpression significantly rescued the reduction in colony formation, migration, and invasion caused by EpCAM silencing in HP14.5-Core cells (Fig. 3K through M). Overall, HCV core induces EpCAM expression across multiple cell types, and EpCAM is functionally required for core-driven proliferation, migration, and invasion in HP14.5 cells.

### HCV core upregulates EpCAM expression by activating the Wnt/β-catenin signaling pathway in HPCs

**Fig 4:**
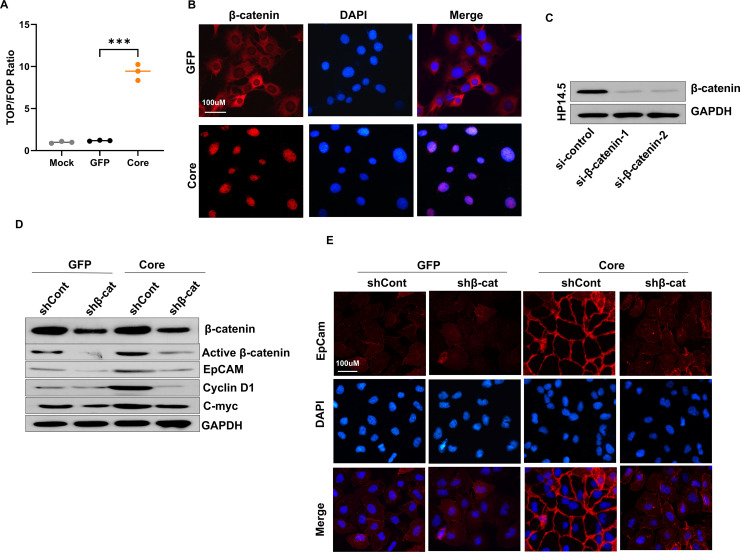
HCV core upregulates EpCAM expression through Wnt/β-catenin signaling in HP14.5 cells. (**A**) TOP/FOP luciferase reporter assay in HP14.5 cells. Cells were transfected, and 16 h after transfection, cells were infected with AdGFP or AdCore (MOI 50) and cultured for 24 h before harvest. Luciferase activity was measured, and results are expressed as the TOP/FOP ratio normalized to Renilla luciferase (*n* = 3). ***P < 0.01 vs GFP control. (**B**) IF staining of β-catenin (red) and DAPI (blue) showed increased nuclear accumulation of β-catenin in HP14.5-Core cells compared with GFP controls (*n* = 3). (**C**) Validation of β-catenin silencing by two independent siRNAs for 48 h (*n* = 3). (**D**) β-Catenin, active β-catenin, EpCAM, cyclin D1, and c-Myc in HP14.5-GFP and HP14.5-Core cells transduced with AdR-shβ-catenin (shβ-cat) or control virus (shCont) were measured (*n* = 3). (**E**) IF results of EpCAM in HP14.5-GFP and HP14.5-Core cells following AdR-shβ-catenin (shβ-cat) or shControl infection (*n* = 3).

The Wnt/β-catenin pathway drives EpCAM expression as a downstream β-catenin/TCF target, thereby sustaining abnormal proliferation, differentiation, and self-renewal of EpCAM^+^ HCSCs (26, 27). To evaluate whether HCV core influences this pathway, we first measured β-catenin activity in HP14.5 cells using a pTOP-Luc reporter. Compared with GFP and mock controls, HCV core expression enhanced β-catenin activity (Fig. 4A). IF analysis further demonstrated that HCV core promoted nuclear accumulation of β-catenin (Fig. 4B). We then used two siRNAs (si-β-catenin-1 and si-β-catenin-2) to transiently silence β-catenin to confirm its role. Both constructs reduced β-catenin protein levels (Fig. 4C), and si-β-catenin-1 was selected for subsequent experiments.

To further confirm the role of β-catenin, HP14.5-GFP and HP14.5-Core cells were transduced with AdR-shβ-catenin (data not shown). In HP14.5-Core cells, β-catenin knockdown with AdR-shβ-catenin reduced active β-catenin and Wnt downstream effectors EpCAM, cyclin D1, and c-Myc (Fig. 4D). Consistently, IF staining confirmed that EpCAM expression was diminished in β-catenin-silenced Core cells compared with controls (Fig. 4E). Taken together, HCV core promotes EpCAM expression in HPCs by activating Wnt/β-catenin signaling, largely via nuclear translocation of β-catenin.

### HCV core interacts with β-catenin to enhance EpCAM expression

**Fig 5:**
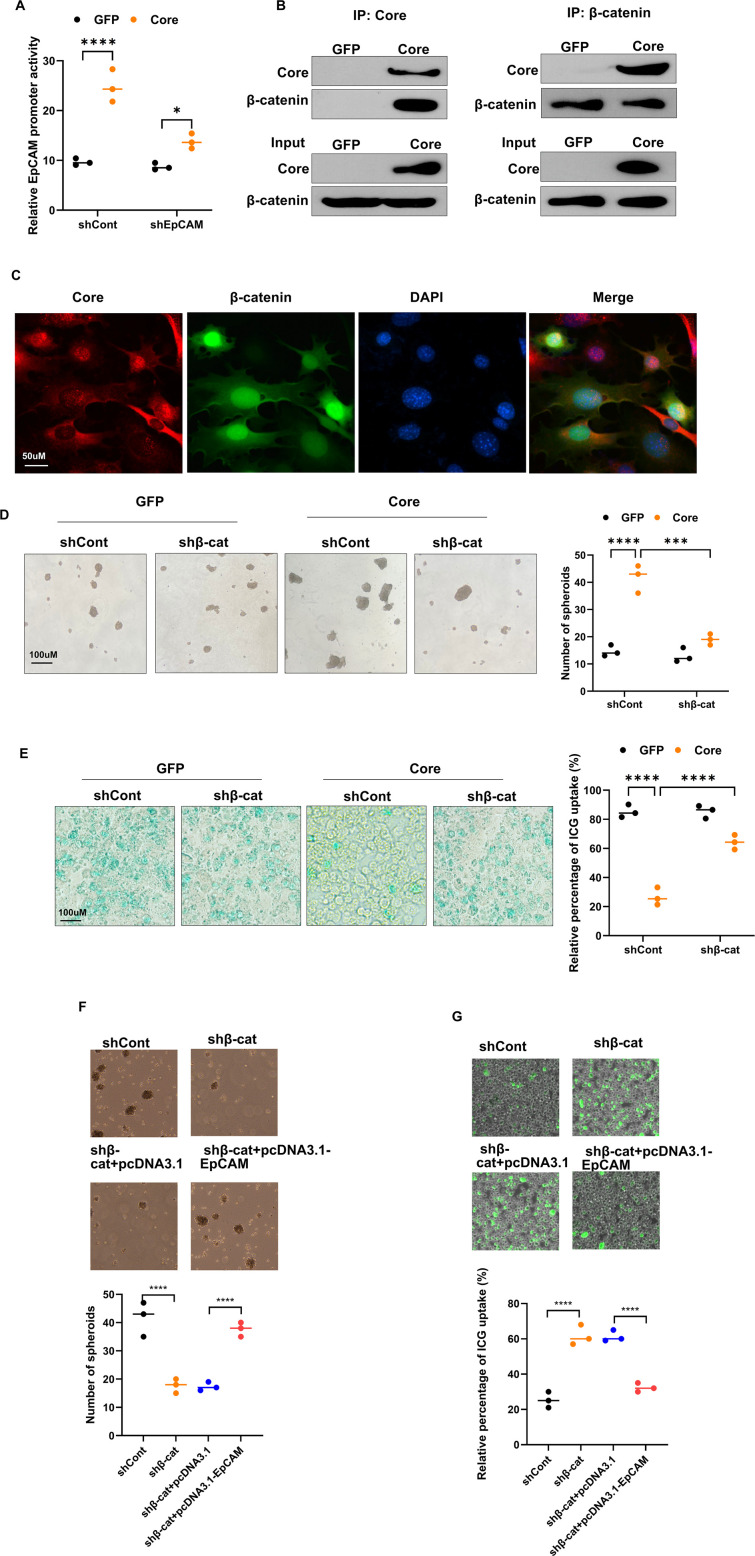
HCV core mediates EpCAM upregulation through interaction with β-catenin and EpCAM overexpression restores cancer stem cell properties in HP14.5-Core cells following β-catenin knockdown. (**A**) Luciferase reporter assay of EpCAM promoter activity in HP14.5-GFP and HP14.5-Core cells transduced with shControl or shEpCAM (*n* = 3). *P < 0.05, ****P < 0.0001. (**B**) Co-IP showed physical interaction between HCV core and β-catenin. Lysates from AdCore-infected HP14.5 cells were immunoprecipitated with anti-core or anti-β-catenin antibodies and probed with the indicated antibodies (*n* = 3). (**C**) IF staining of HCV core (red), β-catenin (green), and DAPI (blue) in AdCore-infected HP14.5 cells (*n* = 3). (**D and E**) Spheroid formation and ICG uptake assay in HP14.5-GFP and HP14.5-Core cells transduced with shControl or shβ-catenin (*n* = 3). ***P < 0.001, ****P < 0.0001. (**F**) Spheroid formation and (**G**) ICG uptake assay in HP14.5-Core cells transduced with shβ-catenin and pcDNA3.1-EpCAM (*n* = 3). ****P < 0.0001.

To test whether EpCAM itself modulates its transcription downstream of HCV core, we measured EpCAM promoter activity in HP14.5-GFP and HP14.5-Core cells after EpCAM knockdown. HCV core strongly increased EpCAM promoter activity under shControl, and this increase was significantly blunted by shEpCAM (Fig. 5A), consistent with a positive feedback loop on EpCAM transcription. To further examine the relationship between HCV core and β-catenin, we performed co-IP assays and confirmed that HCV core was physically associated with β-catenin (Fig. 5B). IF analysis supported these findings, showing that while HCV core predominantly localized to the perinuclear cytoplasm, nuclear colocalization of HCV core and β-catenin was also observed (Fig. 5C). Functionally, β-catenin knockdown in HP14.5-Core cells impaired spheroid formation capacity (Fig. 5D) and restored ICG uptake (Fig. 5E), both indicators of reduced stem-like behavior. Furthermore, EpCAM overexpression reversed the inhibitory effect of β-catenin silencing on spheroid formation and abolished the enhancement of ICG uptake induced by β-catenin silencing (Fig. 5F and G). Overall, HCV core forms a complex with β-catenin to drive EpCAM expression and promotes HPC differentiation into HCSCs.

## DISCUSSION

HCV infection is a major cause of HCC. During long-term infection, HCV interacts with host signaling pathways, driving abnormal proliferation and impaired differentiation of hepatic cells, events central to hepatocarcinogenesis (44). A recent study demonstrated that HCV-infected HPCs acquire HCSC characteristics (45). Our study extends these observations by showing that the HCV core protein promotes the differentiation of HPCs into HCSCs through EpCAM upregulation. Mechanistically, HCV core enhances nuclear translocation of β-catenin, activates the Wnt/β-catenin pathway, and physically interacts with β-catenin to form a coactivator complex. This complex drives EpCAM expression, supporting a model where β-catenin functions as a critical bridge between HCV core and EpCAM in malignant transformation.

CSCs are recognized as drivers of tumor initiation, metastasis, chemoresistance, and heterogeneity (46). Accumulating evidence suggests that HPCs with impaired differentiation serve as a reservoir for HCSCs and that viral proteins contribute to this process (47). In HBV-associated HCC, HBx induces HPC differentiation into HCSCs (48), while HCV NS5A cooperates with TGF-β and Ras signaling to promote EMT and tumorigenesis (49). Furthermore, our findings support previous observations that HPCs infected with HCV acquire HCSC-like characteristics (45, 50). EpCAM, CD133, and CD90 mark both hepatic and cancer stem cells (51, 52). EpCAM, normally seen in developing but not mature hepatocytes (53), is re-expressed in HCC, where it predicts poor survival (54). EpCAM^+^ cells from AFP^+^ HCC tissues further show CSC-like behavior (55, 56). We showed that EpCAM knockdown in HP14.5 cells suppressed proliferation, migration, and invasion, indicating that HCV core may drive the conversion of HPCs into HCSCs through EpCAM upregulation. In line with these reports, we show that HCV core increases the expression of EpCAM, CD133, and CD90 and that EpCAM knockdown suppresses colony formation, migration, and invasion in HPC-derived cells. These findings highlight EpCAM as a functional mediator of HCV core-driven transformation.

Clinical evidence also supports this model. In patient samples, EpCAM expression regulated by Wnt/β-catenin signaling correlates with HCSC growth and poor prognosis (42). More broadly, pathways including Wnt/β-catenin, Hedgehog, TGF-β, and IL-6/STAT3 have been implicated in CSC maintenance (57–59). Among them, Wnt/β-catenin signaling is particularly relevant, as it governs stem cell self-renewal and is aberrantly activated in over half of HCC cases (60–62). Our data add to this body of work by showing that HCV core induces nuclear translocation of β-catenin and enhances recruitment of β-catenin/TCF4 to the EpCAM promoter, thereby increasing EpCAM expression.

Structurally, β-catenin contains N-terminal, central Armadillo (ARM) repeat, and C-terminal domains, enabling interactions with multiple partners including TCF, E-cadherin, and APC. Viral proteins also exploit these interactions. For instance, HIV-1 Nef interacts with β-catenin (63) and with HCV core, enhancing NF-κB activation (64). Consistent with this, we show that HCV core binds β-catenin, which may stabilize its nuclear localization. Prior studies demonstrated that phosphorylation events, such as PKA-mediated phosphorylation at Ser675, enhanced stability and transcriptional activity (65–67). It is possible that HCV core similarly stabilizes β-catenin although the precise interaction domains remain to be defined.

One limitation of this study is that our findings were primarily derived from cell line and mouse models, and validation in patient-derived tissues is still required. In addition, the precise molecular domains through which HCV core interacts with β-catenin remain undefined and warrant further investigation.

In summary, our work identifies the HCV core protein as a driver of HPC-to-HCSC differentiation during HCV-related hepatocarcinogenesis. By forming a complex with β-catenin, HCV core activates Wnt/β-catenin signaling, increases EpCAM expression, and promotes malignant features such as spheroid formation and invasive growth. These findings provide new mechanistic insights into HCV-induced HCC and suggest that disrupting the HCV core-β-catenin-EpCAM axis may represent a therapeutic option for patients with HCV infection.

## Data Availability

Readers are requested to contact Professor Dan Nie for any questions regarding for the manuscript.
